# The keys to success: personality traits and mental strategies are associated with success indicators in special forces selection

**DOI:** 10.3389/fpsyt.2026.1774452

**Published:** 2026-03-12

**Authors:** Daniela da Silva, Pablo Mair, Karl-Heinz Renner, Ladina Throne, Ulrich Wesemann

**Affiliations:** 1 Kommando Spezialkräfte, Abteilung Psychologischer Dienst, Calw, Germany; 2 Charité – Universitätsmedizin Berlin, corporate member of Freie Universität Berlin and Humboldt-Universität zu Berlin, Berlin, Germany; 3 University of the Bundeswehr Munich, Department of Psychology, Munich, Germany; 4 Bundeswehrkrankenhaus Berlin, Psychotraumazentrum der Bundeswehr, Berlin, Germany; 5 Department of Psychiatry, Psychotherapy and Psychotraumatology, Bundeswehrkrankenhaus Berlin, Berlin, Germany

**Keywords:** mental skills, mindset, military personnel, special forces, SOF, success, performance

## Abstract

**Introduction:**

The selection process for the German Armed Forces Special Forces places extreme physical and psychological demands on candidates. The aim of this study was to examine associations between psychological traits and indicators of success in the multi-stage potential assessment procedure (PFV) and to explore individual resources and mental strategies reported by applicants under high stress. Specifically, we examined whether (H1) conscientiousness and cognitive performance are associated with PFV performance, (H2) successful applicants report self-regulation strategies (e.g., goal setting, self-talk, mental visualization, arousal control) and psychological protective factors (e.g., perseverance/grit, optimism, value-based meaning orientation) more frequently, and (H3) resilience is associated with marksmanship performance during training.

**Methodology:**

A sequential explanatory mixed-methods design was applied. In the quantitative part, a secondary analysis of psychological PFV data (N = confidential) was carried out. Associations between personality traits and success indicators (internal PFV ranking; external shooting performance) were examined using Spearman rank correlations. In the qualitative part, guided interviews with applicants (N = confidential) were analyzed using qualitative content analysis following a deductive–inductive approach.

**Results:**

In line with H1, conscientiousness (r = −.74, p <.05) and cognitive performance (r = −.72, p <.05) showed significant associations with better PFV performance. In line with H3, resilience was significantly associated with better shooting performance during training (r = .70, p <.05). The qualitative analysis revealed recurring self-regulation strategies and psychological protective factors that were descriptively reported more frequently by successful participants.

**Conclusion:**

The findings indicate that specific personality traits and self-regulation-related resources are associated with performance-related indicators within the PFV context. The integration of quantitative and qualitative results provides exploratory insights relevant for psychological assessment and mental preparation in a high-performance military setting. Given the study design, conclusions regarding prediction or causality are not warranted; future longitudinal studies with larger samples are needed to examine robustness, generalizability, and temporal stability of the observed associations.

## 1 Introduction

Special forces are considered the most elite military units. They usually consist of highly qualified and motivated individuals who are capable of operating in mentally and physically demanding environments with a high level of commitment. Selection procedures for these units are internationally described as particularly demanding and highly selective; and the literature consistently reports high dropout rates of around 80% (1).

While classic selection procedures in occupational and organizational psychology often rely on standardized testing methods with high reliability and validity (2), the military context—especially in the case of special forces—poses additional challenges. The German Armed Forces’ Special Forces also seeks individuals who are physically, mentally, and cognitively highly resilient and who can act reliably, purposefully, and stably under extreme conditions (3). This aptitude potential is systematically assessed in the KSK in a multi-stage potential assessment procedure (PFV). In addition to cognitive performance and professional motivation, aspects such as emotional stability, resilience, perseverance, and mental flexibility come to the fore (4, 5). Added to this is the specific stress situation of the selection process itself, in which applicants must perform over several days under sleep deprivation, physical exhaustion, and constant pressure to perform.

Previous studies have demonstrated the importance of specific personality traits such as conscientiousness, emotional stability, and cognitive performance for training success in the KSK context (6). Initial empirical evidence from the revised PFV (7) show that conscientiousness and resilience in particular are closely associated with successful placement in the selection process and with objective training performance. At the same time, high dropout rates and individual differences in perseverance remain to be explained: Why do cognitively suitable candidates also fail? What psychological factors make the decisive difference?

Although psychometric data on psychological traits associated with success in military selection are increasingly available (e.g., 6, 8), less research has been done to date with regard to mental strategies, motivational attitudes, and subjective resources which may be relevant for successful completion of such procedures [see (9) for an exception]. In particular, the conscious management of mental states—for example, through self-talk, visualization, or goal-setting strategies—could have a significant impact on psychological resilience under extreme conditions.

By combining quantitative performance and personality data with qualitative interview statements, we aim to contribute to a more comprehensive understanding of psychological traits and resources associated with successful completion of the PFV. The chosen mixed-methods approach allows us to integrate both objectively measurable psychological traits (e.g., cognitive performance, emotional stability) and subjective perspectives on mental strategies and motivation. The aim is not to identify causal predictors, but rather to examine patterns of association between psychological traits and indicators of success in the selection process and to derive descriptive implications.

This should contribute to the evidence-based further development of selection procedures for military special forces – particularly with regard to the question of what successful candidates do differently. Against the backdrop of growing demands on resilience, decision-making ability, and mental toughness, providing empirically grounded insights into psychological traits and strategies that are more frequently observed among successful candidates.

Based on theoretical and empirical prior knowledge, this study derives three central research questions:

• H1 (Traits & Cognition): Higher levels of conscientiousness and cognitive performance are positively correlated with success in the KSK’s PFV (internal ranking). (2–7)
• H2 (Strategies & Resources): Successful applicants report a higher frequency of self-regulation strategies (e.g., goal setting, self-talk, mental visualization, arousal control) and the presence of psychological protective factors (e.g., perseverance/grit, optimism, value-based meaning orientation) than unsuccessful applicants.
• H3 (Resilience & Performance): Resilience is positively related to shooting performance in military training, which is examined as an external performance-related criterion.

These questions will be answered using a sequential explanatory mixed-methods design (10). The theoretical framework and relevant psychological constructs will be explained below.

### 1.1 Theoretical background

Psychological aptitude research in a military context has long been concerned with the question of which individual psychological traits are associated with successful performance in selection procedures, training, and subsequent deployments. Empirical findings show that, in addition to cognitive abilities, personality traits are closely related to performance outcomes, professional development, and training-related indicators (11, 12). In the field of military special forces, it has also been demonstrated that demographic, psychological, and physiological characteristics have likewise been shown to be systematically associated with outcomes in highly selective selection processes (13).

Although not tailored specifically to the special forces domain, these works provide a robust evidence base for the relevance of these characteristics in high-performance environments. Application-oriented investigations in military settings further highlight the role of resilience, emotional stability, and mental flexibility in coping with extreme demands (4, 14).

The strain profile in procedures such as the PFV differs markedly from civilian assessments: time pressure, sustained physical load, social uncertainty, and reduced orientation demand pronounced self-regulatory and stress-processing capacities (15). Classical intelligence tests or structured interviews often capture these reactions only inadequately, necessitating consideration of additional psychological factors.

#### 1.1.1 Personality and performance: big five and beyond

Within personality psychology, the Big Five have been established as a theoretically and empirically robust framework for assessing stable interindividual differences (16). A meta-analysis by Salgado (6), based on job performance across various European contexts—including military—shows that conscientiousness and emotional stability (the opposite pole of neuroticism) show associations with job performance outcomes. Importantly, these associations remain observable even when cognitive ability is statistically controlled.

In high-performance military settings such as the KSK, these dimensions are especially relevant as well: Conscientious individuals typically exhibit goal orientation, self-discipline, and perseverance—qualities that matter in lengthy and demanding selection procedures (17). Emotional stability is associated with lower stress reactivity and greater tolerance for uncertainty and performance pressure (18).

Complementing the Big Five taxonomy, the construct of hardiness has been shown to be closely related to adaptation and performance in military contexts (19). Hardiness is understood as a triad of commitment, control, and challenge—an attitude that interprets stressful situations as manageable and conducive to growth (19). Empirical findings indicate that higher levels of hardiness are associated with better training-related outcomes (4).

#### 1.1.2 Motivation, volition, and grit

Beyond stable personality traits, motivational constructs are increasingly central to understanding successful performance under extreme conditions. Motivation encompasses the direction, intensity, and persistence of goal-directed behavior (20). In selection procedures such as the PFV, which are characterized by substantial deprivation, not only wanting but also persevering—i.e., volition—may be particularly relevant (21).

A central construct in this context is grit, defined as the combination of passion and perseverance for long-term goals (22). In studies with West Point cadets, grit is positively associated with successful completion of basic military training and predicted performance beyond intelligence and fitness measures (22). Grit relates positively to conscientiousness; the question of its incremental contribution relative to classic personality measures is discussed controversially in the literature (23).

Also central is self-efficacy (24), understood as the subjective belief in one’s ability to master difficult tasks through one’s own efforts. Studies in military contexts report that higher self-efficacy is associated with better coping with stress, greater frustration tolerance, and more stable performance under pressure (25, 26).

#### 1.1.3 Mental strategies: self-regulation under strain

In addition to stable dispositions such as personality and motivation, conscious mental strategies are increasingly coming into focus, which individuals use to specifically control their attention, emotions, and motivation under highly stressful conditions. (27). Such strategies are considered trainable and are therefore particularly relevant for selection and preparation.

Successful candidates in high-performance environments frequently report using self-talk, goal-directed mental planning, imagery, and focusing techniques to stabilize performance under pressure (28, 29). Originally developed in competitive sport, these techniques have increasingly been transferred to military high-strain contexts to foster psychological performance there (30).

A qualitative study examining mental strategies among special forces applicants shows that PFV finishers report the use of self-regulatory strategies more frequently than non-finishers (dropouts, i.e. applicants who voluntarily discontinued participation in the PFV).

Most frequently reported were:

• Positive self-talk (“You can do this, step by step”),
• Mental visualization of the goal (e.g., imagining arriving at the end of the week),
• Emotional reappraisal (“This is only a test of my willpower”),
• External goal commitment (thoughts of family, comrades, values).

These strategies can be situated within Kuhl (21) model of self-regulation, which distinguishes volitional action control from motivational impulse control. Especially under strain, the ability to align action impulses with superordinate goals may be particularly relevant.

### 1.2 Theoretical framing: stress models and resilience

Psychological success factors in the PFV can also be interpreted in light of stress models. The transactional model of stress by Lazarus and Folkman (31) emphasizes that strain is not solely objective but depends decisively on subjective appraisal processes: whether a situation is experienced as “threat” or “challenge” shapes emotional and physiological responses.

In this context, resilience—the capacity for positive adaptation despite adverse circumstances—plays a central role. Resilience is not a fixed personality trait but a dynamic process grounded in protective factors such as self-efficacy, social support, and emotion regulation (32). Studies with elite soldiers show that more resilient individuals more often employ adaptive coping and less often resort to maladaptive avoidance (33).

These processes can be linked to psychological flexibility—the capacity to act appropriately despite internal obstacles such as fear, stress, or exhaustion (34). Especially in selection procedures, where routines are scarce and novel challenges dominate, psychological flexibility may represent a relevant resource.

### 1.3 Interim conclusion: a hypothetical model of success factors in the PFV

Based on the findings presented, a hypothetical model of psychological traits associated with successful performance in the PFV can be outlined that integrates stable traits (e.g., conscientiousness, emotional stability), motivational constructs (e.g., grit, self-efficacy), and dynamic strategies (e.g., self-talk, goal commitment). It is plausible that these dimensions do not operate in isolation but interact—for example, high self-efficacy may increase the likelihood of deploying mental strategies, or emotional stability may modulate stress appraisal.

The aim of the present study is to examine these factors empirically, to analyze their associations, and to derive descriptive indicators of psychological traits related to differences between successful and unsuccessful candidates. The following Methods section outlines the specific procedures within the sequential explanatory mixed-methods design.

## 2 Methods

The Special Forces Command’s Potential Assessment Procedure (PFV) consists of psychological, physical, and military components, which are weighted equally at the outset. This article focuses exclusively on the psychological components of the PFV; the physical and military elements of the procedure are not explored in depth for reasons of thematic focus, but are an integral part of the overall process. Based on a requirements analysis, key aptitude psychological traits were defined that are considered critical to the success of special forces in their specific mission. A multi-method approach is used to assess these psychological traits, consisting of computer-based performance tests, standardized questionnaires, semi-structured interviews, and systematic behavioral observations in order to avoid mono-methodological biases and increase the validity of the assessment (35, 40).

The PFV is structured in several stages. In phase 1, cognitive abilities and minimum physical standards are tested to ensure basic trainability. Only candidates who meet these minimum requirements are considered further. In phase 2, a reference group-oriented selection process with broad psychological diagnostics takes place. Here, personality traits and performance- and behavior-related criteria are assessed by psychologists and military trainers. For applicants to the officer career path, phase 3 follows with a focus on leadership behavior under stress. The final ranking serves as the basis for the selection decision and takes into account psychological as well as physical and military performance indicators.

### 2.1 Study design

This investigation follows a sequential explanatory mixed-methods design (10), in which quantitative data on psychological suitability and success in selection and training within the PFV at the Bundeswehr’s KSK are analyzed first. Building on the quantitative results, a qualitative deepening is conducted through content-analytic evaluation of interview data to better understand subjective determinants and mental strategies of successful and unsuccessful candidates. The goal is to integrate correlational findings with subjective perspectives with individual patterns of experience and action, thereby developing a more comprehensive understanding of the psychological key factors in the selection process.

### 2.2 Quantitative part

#### 2.2.1 Sample and data basis

Quantitative data were collected as part of real KSK selection procedures from 2023 to 2025. The analysis included only candidates who completed the PFV in full and under standardized conditions. For reasons of confidentiality and operational security, no absolute sample sizes or personal psychological traits can be reported. This restriction is a necessary measure to prevent inferences about personnel structures in the special forces domain and is noted as a limitation in the discussion.

The quantitative results of this work are based on a secondary analysis of psychological data from the PFV of the KSK that were previously published as part of an earlier analysis (7). The focus here is on associations between selected psychological traits and success in the selection process, operationalized via an internal ranking and an objective external criterion (marksmanship performance during subsequent training).

For reasons of operational security, neither absolute sample sizes nor approximate sample size ranges are reported, as even aggregated information could permit inferences about personnel structures within the special forces domain.

#### 2.2.2 Assessed personal psychological traits

The following psychological, cognitive, and behavioral psychological traits were systematically recorded as part of the PFV:

• Cognitive ability

• Information processing capacity

• Attentional/concentration capacity

• Social competence

• Conscientiousness

• Emotional stability

• Resilience

• Tolerance of failure

• Decisiveness (e.g. VUCA)

• Personality and behavioral stability

The assessment in the PFV is based on a combination of various psychologically established methods. These include internally and externally validated cognitive performance tests, standardized personality questionnaires, and structured interviews. In addition, psychological behavioral observations are carried out in realistic stress situations, which have a high ecological validity. The final assessments are carried out by psychologists who are specially trained in the aptitude assessment of military selection processes. For reasons of confidentiality, the individual instruments cannot be disclosed in detail here; for a more comprehensive description of the structure and organization of the PFV, please refer to da Silva et al. (7).

#### 2.2.3 Success criteria

To quantitatively examine performance-related outcomes within the selection and training context, two criteria were considered:

• PFV ranking: Final placement at the end of the selection process served as the primary indicator of selection outcome. The ranking reflects an aggregated evaluation by the selection panel and integrates psychological assessments as well as physical and military performance components.
• Marksmanship during basic training: Shooting performance during subsequent basic training was examined as a downstream, task-specific external performance indicator. This measure represents an objective and standardized assessment of shooting proficiency within a clearly defined military task. While marksmanship performance constitutes a practice-relevant and operationally meaningful outcome, its interpretation is limited to this specific performance domain; broader inferences regarding general training suitability or long-term performance are therefore made cautiously.

#### 2.2.4 Statistical analysis

The statistical analysis comprised exclusively bivariate correlations (Spearman rank correlations) to examine associations between psychological traits and PFV success criteria. The focus was specifically on conscientiousness, emotional stability, and resilience, analyzing their associations with the internal selection ranking as well as an external performance indicator (marksmanship during training). Statistical significance was set at p <.05.

Given the exploratory nature of the study, the limited and non-disclosed sample size due to operational security constraints, and the focus on hypothesis-guided associations, no formal correction for multiple testing was applied. Consequently, reported p-values are interpreted descriptively rather than as confirmatory evidence. The results should therefore be considered as indicative patterns of association that require replication in larger samples using multivariable and confirmatory analytical approaches.

### 2.3 Qualitative part: interview study

The qualitative results are based on semi-structured interviews with candidates in the KSK’s PFV in 2025. The interviews were conducted in the week of phases 2 and 3 of the selection procedure and included both candidates who completed the process in full (finishers) and those who ended it early by self-withdrawal or removal (non-finishers). Assignment to these groups was made retrospectively based on the outcome of the procedure. The qualitative component aimed to explore subjective experiences, mental strategies, and motivational resources reported by candidates that are associated with perseverance and completion or discontinuation of the PFV.

#### 2.3.1 Methodological approach

Analysis was conducted using qualitative content analysis according to Kuckartz (36, 37). A deductive–inductive procedure was employed: top-level and sub-categories were first derived from the pertinent literature as well as from the interview guide and were expanded and differentiated over the course of coding (**Table 1**).

**Table 1:** Categories and subcategories.

Category	Subcategory
Motivation & Goal Commitment	Intrinsic Motivation
	Extrinsic Motivation
	Goal Clarity/Goal Commitment
	Preparatory Actions/Implementation Motivation
Mental State & Self-Efficacy	Belief in One’s Mental Strength — Self-Efficacy & Locus of Control
	Change in Self-Concept
	Mastery Experiences (Self-Efficacy Experiences)
	Perceived Relevance of Mental Strength/Strategies
Mental Strategies for Coping with Strain	Goal Setting
	Mental Imagery (Mental Rehearsal)
	Self-Talk / Inner Dialogue
	Arousal Control
	Strategy Shift / Situation Adaptation
	Intuitive/Non-Strategic Coping
Psychological Protective Factors & Grit	Grit/Perseverance Despite Setbacks or Boundary Experiences
	Positive Reattribution / Reframing
	Optimism / Hope
	Moral Meaning / Values Basis
Reasons for Withdrawal	Physical Exhaustion / Injury
	Mental Overload / Psychological Strain
	External Factors / Personal Reasons
	Psychological Risk Factors
	Emotional State After Withdrawal
	Applicants’ Subjective Theories of Relevant Success Factors and Risk Factors (for Withdrawal)
Social & Biographical Background	Family Support / Private Situation
	Military background / previous experience

The final categories included, among others, mental strategies, motivational factors, protective and risk factors, and experiences of strain.

#### 2.3.2 Sample and data collection

In addition to the quantitative results, interview data from the PFV of 2025 was evaluated qualitatively in order to gain additional insights into individual processing processes. The interviews took place in the same week as PFV phases 2 and 3 and were conducted with participants who either

• successfully completed the selection process to the end (finishers), or
• ended it early through self-withdrawal or removal (non-finishers).

Candidates who discontinued the procedure at earlier stages are not represented.

The selection of interview partners was purposeful along these two groups to enable a contrastive analysis of psychological strategies and motivational differences.

#### 2.3.3 Interview guide

The interviews followed a semi-structured guide aimed at the open exploration of individual experiences during the PFV. The full guide is available upon request.

#### 2.3.4 Analysis approach

The semi-structured interviews with PFV candidates were conducted after phases 2 and 3 and analyzed using qualitative content analysis according to Kuckartz (36). This procedure allows the integration of deductive, theory-based and inductively derived categories, thereby systematically considering both predefined psychological constructs and newly emerging content-based phenomena. The analysis focused specifically on identifying mental strategies, motivational–cognitive influencing factors, and individual protective and risk factors associated with successful or unsuccessful completion of the selection procedure.

This approach enables a structured linkage of theory-driven and data-driven categories. Development of the category system followed a deductive–inductive procedure: it began with deductively developed top-level and sub-categories derived both from relevant theoretical concepts (e.g., self-regulation, motivation, resilience) and from the thematic foci of the interview guide.

Over the course of coding, these categories were iteratively revised. Recurring patterns, mental strategies, or psychological traits that went beyond the original system were systematically added as new sub-categories or used to differentiate existing ones. Categories that did not demonstrate sufficient distinctiveness were merged. The aim of the analysis was to delineate both cross-group commonalities and psychological traits differences between finishers and non-finishers, thereby identifying psychologically relevant success factors in the selection process.

## 3 Results

### 3.1 Quantitative findings

#### 3.1.1 H1 associations between personality traits, cognitive performance, and internal PFV ranking

Final placement at the end of the PFV served as the primary indicator of selection outcome. This internal ranking reflects an aggregated evaluation by the selection committee based on psychological, physical, and military performance components; lower ranking values indicate better placement. During Phase 1 of the PFV, cognitive performance (r = .94, p <.05) and concentration capacity (r = –.62, p <.05) showed significant associations with final ranking position.

Across Phase 2 and the overall selection process, conscientiousness was significantly associated with PFV ranking (r = –.74, p <.05), with higher conscientiousness corresponding to better placement. Cognitive performance likewise demonstrated a significant association with PFV ranking (r = –.72, p <.05). Other assessed psychological traits (decisiveness, concentration capacity, resilience) showed non-significant trends toward associations with selection outcomes.

An overview of all examined bivariate associations is provided in **Table 2**.

**Table 2:** Intercorrelations in the PFV and the external criterion (shooting performance).

Variable	*M*	*SD*	Shooting performance	Cognitive ability	Attentional / Concentration capacity	Information processing capacity	Ranking	Tolerance of failure	Resilience	Personality and behavioral stability	Conscientiousness	Emotional stability	Decisiveness (VUCA)	Cognitive ability	Attentional / Concentration capacity	Social competence	Ranking
Phase I																	
Shooting Performance	179	13,3		-.1	-.23	.32	.17	-.37	.7*	-.09	-.23	-.3	-.13	-.52	.5	-.26	.24
Cognitive ability	2,65	0,74			-.53	-.1	.94	-.3	-.39	-.12	-.37	0	.31	-.03	-.2	.21	.49
Attentional / Concentration capacity	63,7	9,96				.6	-.62	.45	-.24	.24	.73*	.5	-.21	.45	.46	-.17	-.83
Information processing capacity	66,7	18,9					-.03	.22	.01	-.09	.28	.5	-.11	-.17	.93	-.44	-.34
Ranking	XX	XX						-.37	-.12	-.15	-.56	0	.11	-.18	-.07	.09	.59
Phase II																	
Tolerance of failure	2,82	0,4							-.32	-.36	.47	.67	-.23	.12	.23	-.56	-.34
Resilience	1,82	0,56								-.32	-.39	-.21	-.54	-.26	.27	-.07	.15
Personality and behavioral stability	2,64	0,5									.06	-.24	.18	.19	-.31	.27	-.24
Conscientiousness	2,38	0,27										.21	.22	.48	.19	.02	-.74
Emotional stability	2,91	0,3											-.51	.38	.36	-.25	-.5
Decisiveness (VUCA)	2,24	0,43												-.27	-.22	.26	.2
Cognitive ability	2,14	0,55													-.34	.58	-.72
Attentional / Concentration capacity	2,41	0,38														-.6	-.16
Social competence	2,35	0,26															-.27
Ranking	XX	XX															

Spearman rank correlations between psychological traits, internal PFV ranking, and shooting performance.

*Spearman’s ρ; p <.05 (reported descriptively); no correction for multiple testing was applied. Sample sizes and ranking descriptives are omitted due to OPSEC constraints.

#### 3.1.2 H3: associations between psychological traits and an external performance indicator

In addition to the internal PFV ranking, associations between selected psychological traits and an external, task-specific performance indicator—marksmanship performance during subsequent basic training—were examined.

Resilience showed a significant positive association with marksmanship performance (r = .70, p <.05), indicating that candidates with higher resilience scores tended to achieve better shooting results during training. No significant associations were observed between marksmanship performance and other psychological traits, including conscientiousness and emotional stability.

All bivariate associations between psychological traits and marksmanship performance are summarized in **Table 2**.

### 3.2 Qualitative findings: interview study

#### 3.2.1 H2: motivation and inner attitude

In addition to specific self-regulation techniques, interview data revealed differences in motivational orientations and inner attitudes reported by finishers and non-finishers. Finishers more frequently described higher-order motivational perspectives that extended beyond situational coping strategies.

Reported themes included:

• strong achievement motivation and clarity of personal goals (e.g., “I knew why I was doing this”)
• a pronounced resistance orientation (e.g., “I didn’t want to fail—no matter how difficult it became”)
• acceptance of pain and deprivation as integral components of the selection process, and
• confidence in one’s capacity to act under strain, conceptually related to self-efficacy (24).

H2 was addressed exclusively through qualitative, exploratory analysis and was not subjected to statistical testing; reported frequencies are descriptive only and do not represent quantitative group comparisons.

These findings are based on qualitative self-reports and are interpreted descriptively. An illustrative overview of reported mental strategies and motivational themes is presented in **Table 3**.

**Table 3:** Mental strategies of finishers and dropouts.

Mental strategies			
Category		Dropouts	Finisher
Goal Setting	Intrinsic Motivation	87,5%	100,0%
	Extrinsic Motivation	25,0%	0,0%
	Goal Commitment high	0,0%	100,0%
	Goal Commitment medium	37,5%	0,0%
	Goal Commitment low	62,5%	0,0%
	Preparation high	25,0%	58,3%
	Preparation medium	62,5%	41,7%
	Preparation low	0,0%	0,0%
Mental Imagery (Mental Rehearsal)		25,0%	75,0%
Self Talk	motivational	12,5%	100,0%
	calming	37,5%	41,7%
	Self critical	12,5%	33,3%
	cognitive focusing	25,0%	83,3%
	inner discussion	62,5%	8,3%
Arousal Control		50,0%	41,7%
Change of Strategy		25,0%	41,7%
Intuitive Coping		62,5%	16,7%
psychological protective factors	Grit	37,5%	91,7%
	positive Reattribution	12,5%	75,0%
	Optimism	25,0%	41,7%
	Moral sense / value basis	0,0%	50,0%

Percentages refer to the presence of statements within the interviews that could be coded into the respective categories. *Dropouts* are applicants who voluntarily discontinued participation in the PFV, whereas *finishers* are those who completed the entire PFV. Percentages do not represent statistical group comparisons.

## 4 Discussion

### 4.1 Discussion of quantitative results

The quantitative analysis examined bivariate correlations between psychological traits and performance-related indicators within the Special Forces Command’s potential assessment procedure (PFV). Findings were obtained for both the internal selection ranking and a task-specific external performance indicator assessed approximately a few months later.

There was a significant correlation between conscientiousness and the internal ranking, with higher scores being associated with better placement. This finding is consistent with earlier studies that have reported associations between conscientiousness and performance-related outcomes (e.g., 6, 23), particularly in military contexts (38). Rather than indicating a causal effect, this association suggests that psychological traits typically linked to conscientiousness—such as persistence, goal orientation, and self-discipline—may be relevant within demanding and prolonged selection procedures.

Cognitive performance was also associated with the selection ranking. This finding aligns with the expectation that cognitive abilities play a supportive role in coping with complex and dynamic selection demands such as those encountered in the PFV (39). Methodological aspects must be considered in this context, as some cognitive variables are directly incorporated into the aggregated ranking, and the observed associations may therefore be partially inflated due to criterion overlap.

Resilience was found to be correlated with shooting performance during training. Applicants with higher psychological resilience tended to perform better in this task-specific training context. This association may reflect the relevance of stress regulation and performance stability under pressure; however, interpretations are limited to this specific performance domain. Other psychological traits, such as emotional stability or decisiveness, did not show significant associations with this external indicator.

The observed association between resilience and marksmanship performance may plausibly be linked to mechanisms related to stress and arousal regulation, attentional control, and coping under evaluative pressure. Higher resilience may facilitate more stable fine-motor execution and sustained attentional focus in high-stress shooting situations, thereby reducing performance decrements under pressure; however, these mechanisms remain hypothetical and cannot be directly examined within the present study.

Overall, the results indicate that conscientiousness and cognitive performance are associated with internal selection outcomes, while resilience is associated with task-specific training performance. Given the correlational design, the exclusive use of bivariate analyses, and the restricted and non-disclosed sample size, conclusions regarding independent predictive effects or causality are not warranted. Future studies using larger samples and multivariable or longitudinal designs are required to further examine these relationships.

In light of the exploratory correlational approach and the absence of correction for multiple testing, the present findings should be understood as hypothesis-generating rather than confirmatory.

### 4.2 Discussion of qualitative results

The qualitative analysis aimed to explore psychological traits and experiences associated with successful or unsuccessful participation in the Special Forces Command’s Potential Assessment Procedure (PFV). The focus was on motivational–cognitive characteristics, mental strategies for coping with stress, and psychological protective and risk factors, as derived from the interview data.

Participants who completed the PFV frequently described the deliberate use of mental strategies to remain functional under extreme conditions. These included positive self-talk (“Just keep going, step by step”), visualization of goal attainment (“I saw myself getting through it in the end”), emotional reappraisal (“This is a test of my strength”), and value-based goal commitment, often related to family, comrades, or personal ideals. Several applicants described a state referred to as “focus mode” or “tunnel vision,” which facilitated sustained concentration and behavioral control. Preparatory actions—both mental and physical—were also frequently reported. In addition, finishers described a stable sense of self-efficacy and perceived control (“I knew I would get through it if I stuck with it”). These patterns can be conceptually linked to established models of self-regulation, such as Kuhl’s theory of action control (1992) or Zimmerman’s model of self-regulated learning (2000).

Participants who discontinued the PFV reported different subjective experiences. They more often described mental overload, intrusive rumination (“All I could think about was giving up”), and doubts regarding their suitability or the meaning of the procedure. Difficulties in emotion regulation under conditions of sleep deprivation, hunger, and social pressure were also reported. External attributions (“The system was unfair”) tended to replace active coping strategies. These descriptions are consistent with an interplay of reduced volitional control, lower psychological flexibility, and diminished perceived control. Psychological flexibility, defined as the capacity to adapt behavior to changing situational demands, is described in the literature as a protective factor for mental health and performance (34).

Enduring psychological resources also differed descriptively between groups. Participants who completed the PFV more frequently reported grit, optimism, positive reappraisal, and value-based goal commitment. In contrast, participants who discontinued participation more often described risk-related patterns such as fatalistic attitudes, low frustration tolerance, or cynical evaluations. These qualitative differences are descriptive in nature and are not based on statistical testing.

In summary, the qualitative findings suggest that finishers are characterized not only by physical or cognitive capacities, but also by the reported use of mental self-regulation strategies and enduring psychological resources such as value orientation and optimism. These findings complement the quantitative associations observed for conscientiousness and resilience by adding a dynamic, process-oriented perspective.

### 4.3 Integration of quantitative and qualitative findings

Integrating quantitative and qualitative findings allows for a more comprehensive understanding of psychological traits associated with performance-related outcomes in the PFV.

The quantitative associations between conscientiousness and cognitive performance with internal ranking are reflected in qualitative descriptions of structured preparation, goal-oriented behavior, and strategic action under stress—patterns commonly associated with conscientious behavior (cf. 6, 23).

With regard to the external performance indicator, the association between resilience and marksmanship performance corresponds with qualitative reports of perceived control, adaptive coping, and emotion regulation under pressure. These descriptions illustrate that resilience may manifest not only as a dispositional characteristic but also through actively maintained coping processes.

Additionally, the qualitative findings point to further psychological resources—such as grit, optimism, and value-based motivation—that extend beyond the constructs captured by the quantitative measures. While these aspects cannot be statistically evaluated within the present design, they may represent relevant complementary resources for understanding perseverance in highly demanding selection contexts.

Taken together, both data sources suggest that performance-related outcomes in the PFV context are associated with a combination of stable personality traits, cognitive abilities, and situational self-regulation processes. The quantitative findings provide evidence of statistical associations, whereas the qualitative findings offer descriptive insights into subjective experiences and potential underlying mechanisms.

### 4.4 Limitations of the study

Despite these insights, several limitations must be considered. Due to confidentiality requirements, the exact sample size and sociodemographic characteristics could not be reported, limiting statistical inference, power estimation, and generalizability. The quantitative analyses included only candidates who completed the PFV, introducing potential selection and survivorship bias and restricting variance. Furthermore, the exclusive reliance on bivariate correlations limits the ability to examine independent effects or control for confounding variables.

The PFV ranking represents an aggregated criterion that includes psychological components also examined in this study, so partial criterion contamination cannot be excluded. The external criterion—marksmanship performance—captures only a narrow, task-specific aspect of military performance and does not permit conclusions regarding broader training success or long-term operational capability.

The study reports a number of bivariate correlations without applying a formal correction for multiple testing. This increases the risk of alpha inflation and chance findings. Given the exploratory design, the restricted and non-disclosed sample size, and operational security constraints, p-values are interpreted descriptively and without claims of confirmatory inference. Accordingly, the reported associations should be interpreted cautiously and require replication in independent samples.

Due to operational security (OPSEC) constraints, neither absolute sample sizes nor approximate sample size ranges can be reported, as even aggregated or ranged information could allow inferences about personnel structures. As a consequence, formal power calculations, precise assessment of effect size stability, and conventional interpretations of p-values are limited. The reported associations should therefore be interpreted cautiously and understood as exploratory patterns rather than confirmatory evidence, requiring replication in independent samples.

The qualitative sub-study relied on retrospective self-reports and a purposive sample that did not aim for theoretical saturation, which may limit transferability and introduce recall bias. Finally, the absence of longitudinal data precludes conclusions regarding the temporal stability or prognostic validity of the observed associations.

### 4.5 Practical implications

Despite these limitations, the findings offer exploratory insights relevant for psychological selection and preparation in military special forces contexts. The observed associations suggest that psychological traits such as conscientiousness and resilience may be relevant markers within demanding selection environments, although they should not be interpreted as independent predictors of success.

The qualitative findings highlight the role of motivational and self-regulatory processes for sustaining effort under extreme conditions. Strategies such as goal setting, self-instruction, visualization, and cognitive reappraisal were frequently reported and may represent trainable skills relevant for preparation and psychological support.

Rather than implying deterministic selection decisions, the results support an integrative framework that considers stable psychological traits, situational self-regulation strategies, and enduring motivational resources. Interventions aimed at strengthening self-efficacy, psychological flexibility, and value-based motivation may contribute to improved coping and reduced attrition during selection and training, while future research should evaluate these approaches using longitudinal and multivariate designs.

## 5 Conclusion

This study examined psychological traits associated with performance-related outcomes in the selection procedure for military special forces using a sequential explanatory mixed-methods design. By combining quantitative performance-related data with qualitative interview analyses, a differentiated profile of cognitive, emotional, and motivational characteristics reported by successful candidates could be outlined.

The findings indicate that conscientiousness and resilience are associated with performance-related indicators in the selection procedure as well as with task-specific performance during subsequent training. In addition, the qualitative results suggest that motivational resources such as intrinsic motivation, goal clarity, and mental preparation are frequently reported in connection with perseverance under extreme strain. In particular, self-regulatory strategies and psychological protective resources—such as grit, cognitive reframing, and values-based meaning orientation—emerged as descriptively relevant in the accounts of candidates who completed the procedure.

Overall, the findings suggest that psychological traits relevant to special forces selection are multidimensional and dynamic. Rather than a single personality trait being decisive, the results point to an interplay of multiple dispositional psychological traits and situational cognitive and motivational processes that are associated with sustained engagement or discontinuation under pressure.

From a practical perspective, these results offer exploratory implications for the evidence-based refinement of selection and training measures. Psychological assessment procedures may benefit from considering motivational and self-regulatory characteristics alongside established trait-based measures. Furthermore, integrating mental competencies—such as self-regulation, goal commitment, and psychological flexibility—into preparatory programs may support candidates in coping with the psychological demands of the selection process.

Future research should employ longitudinal designs to examine the temporal stability and developmental trajectories of psychological resources among special forces personnel. Such studies could also clarify to what extent specific mental strategies are associated not only with short-term selection-related outcomes but also with longer-term training performance and psychological health during deployment.

## Data Availability

The original contributions presented in the study are included in the article/supplementary material. Further inquiries can be directed to the corresponding authors.
